# A Case Study and Literature Review of Local Anesthetic Systemic Toxicity During Placement of a Dual-chamber Pacemaker

**DOI:** 10.19102/icrm.2024.15013

**Published:** 2024-01-15

**Authors:** Sara King, Perry Nystrom, Jonathan Wajert, Mindy Ferguson

**Affiliations:** 1 Dayton VA Medical Center, Dayton, OH, USA

**Keywords:** Local anesthetic toxicity syndrome, lidocaine, pacemaker implantation

## Abstract

Local anesthetics are commonly deployed for a variety of medical procedures across many disciplines. Systemic toxicity is rarely seen in clinical practice, and quick recognition and how to manage this syndrome are crucial. The development of systemic toxicity is influenced by the site of administration, the type of anesthetic used, and the total dose administered. Local anesthetic systemic toxicity (LAST) syndrome is used as a diagnosis to encompass the cardiovascular and pulmonary adverse effects associated with the intradermal and subcutaneous use of local anesthetics—in our case, lidocaine. We present a case of a 37-year-old man who experienced dysarthria, bilateral arm shaking, and sinus tachycardia following the administration of 70 mL of lidocaine 2% during surgery for dual-chamber pacemaker placement. While some form of allergic reaction remained a possibility, the strongest clinical correlation and diagnosis were attributed to LAST.

## Introduction

Local anesthetic systemic toxicity (LAST) often results in various clinical presentations, though many cases begin with central nervous system (CNS) excitation manifesting in the elevation of vital signs and seizure-like symptoms. Although some risk factors for the development of LAST syndrome are recognized, a complete understanding of patient risk and symptom expression remains elusive.^1^

Use of local anesthetics during the outpatient placement of a pacemaker is commonly accepted as safe and effective analgesia.^2,3^ Benefits include few side effects and limited sedation prior to implementing postoperative pain control.

This case study evaluates symptom progression during dual-chamber pacemaker placement in a patient who developed stroke-like symptoms after subcutaneous lidocaine administration.

## Case presentation

### Patient condition

A 37-year-old man, weighing 73.3 kg, presented for the implantation of a dual-chamber pacemaker for the treatment of long sinus pauses.

As a premedication for pacemaker placement, the patient received 20 mL of subcutaneous lidocaine 2% in addition to 50 μg of fentanyl and 2 mg of midazolam intravenously. During the procedure, vein entry was successful, but the guidewire would not advance, and so vascular access was attempted several times in the upper chest, during which the patient received a total of 70 mL of subcutaneous lidocaine 2%.

The patient subsequently developed dysarthria and bilateral arm shaking in addition to sinus tachycardia with rates up to 120 bpm and a systolic blood pressure of 160 mmHg. Right-sided symptoms resolved quickly but left-sided symptoms persisted. Due to concern for a possible allergic reaction, an intravenous push of 25 mg of Benadryl (Johnson & Johnson, New Brunswick, NJ, USA) in addition to an intravenous push of 2.5 mg of metoprolol was given. Blood pressure and heart rate returned to ±20% pre-procedural levels, but sensory symptoms continued. The procedure was aborted and the patient was directly admitted to the intensive care unit (ICU) for a closer stroke/transient ischemic attack workup with neurology consultation.

A Holter monitor had been placed in 2016 after a single episode of syncope during heavy exertion, which is when the patient’s sinus pauses were first noticed. He was taking metoprolol for hypertension at that time, which was continued. He has complained of intermittent heart racing and fluttering in addition to occasional dizziness with exertion since 2016. He had completed two sleep studies, both of which were negative for sleep apnea, although restless leg syndrome was noted.

He underwent electroencephalographic testing in 2022 secondary to feelings of light-headedness during activity. The electroencephalogram was negative for seizure activity, but he was noted to have pauses up to 4 s long on cardiac monitoring, and he was subsequently monitored via a Zio patch (iRhythm, San Francisco, CA, USA). During Zio patch monitoring, 69 pauses were noted, the longest of which was 11.8 s. The pauses primarily occurred at nighttime and were asymptomatic.

The patient also had a history of traumatic brain injury with loss of consciousness (2014), hypertension, gastroesophageal reflux disease, anxiety, and chronic headaches. Finally, he had no known allergies.

The patient was on 50 mg of metoprolol daily pro re nata (PRN) for hypertension, 150 mg of bupropion extended-release (ER) daily, a 10-mg tab of rizatriptan benzoate (1 tablet PRN for headache), 300 mg of gabapentin (one capsule twice daily for nerve pain PRN), 50 μg of cholecalciferol daily, 40 mg of famotidine twice daily for gastroesophageal reflux disease, and 750 mg of divalproex ER daily.

### Intensive care unit

Upon arrival to the ICU, clinical findings included left-sided facial palsy, slurred speech, left-sided hemiparesis graded as 2/5 according to the Medical Research Council scale for muscle strength, and left hemihypesthesia. The internal medicine team evaluated the patient upon arrival to the ICU, where they awarded the patient 8 points on the U.S. National Institutes of Health (NIH) Stroke Scale and ordered a non-contrast head computed tomography exam, which was negative for any acute abnormalities. Computed tomography angiography of the head/neck showed no evidence of intracranial stenoses or occlusions.

Upon evaluation by the neurology team approximately 90 min later, the patient’s symptoms were much improved. His repeat NIH scale score was 3 points (stratified as 2 points for extremity drift and 1 point for sensory change) and subsequently dropped to 1 point for persistent left-sided sensory disturbances. The right-sided sensory disturbances had completely resolved at this point. Four hours post-symptom development, the patient scored 0 points on the NIH scale and felt that his symptoms had totally resolved.

The patient returned to the operating room the next day for pacemaker placement under general anesthesia. The placement was successful, with no recognized complications. The patient returned to the ICU for an additional day of monitoring and was subsequently discharged home.

### Differential diagnosis

There were several diagnoses in our differential. The potential for acute lacunar infarction and/or cerebral air embolism was based on stroke symptoms present, including left-sided numbness and weakness, as well as mental status changes. Ultimately, non-contrast magnetic resonance imaging of the brain ruled out the possibility of stroke.

A syncopal or anxiety-provoked event was possible. Based on the overall constellation of symptoms, including dizziness, feeling of passing out, generalized weakness, and a robust history of anxiety, a white coat syndrome could have arisen. However, this was unlikely due to the unusual presentation of symptoms, including acute-onset slurred speech and one-sided persistent weakness.

An allergic reaction was also part of our differential due to the sudden symptom onset. The initial impression from the interventionalist noted that the symptoms started after the administration of routine procedural medications. The medications were given at approximately 9:30 a.m., and stroke-like symptoms first occurred at 10:15 a.m. Please note that the patient received additional doses of subcutaneous lidocaine 2% throughout the procedure.

LAST was at the top of our differential. Symptoms started after a subcutaneous administration of 70 mL of lidocaine 2%. The patient received a total of 1400 mg of lidocaine (70 mL × 20 mg/1 mL), which exceeds the recommended limit of 366.5 mg of lidocaine (5 mg/kg × patient’s current weight of 73.3 kg).^4–6^ Stroke-like symptoms are a common manifestation of LAST.

### Pharmacology

The mechanism for LAST has historically been difficult to establish due to the highly variable clinical presentation. Most theories summarizing the associated CNS and cardiovascular (CV) toxicities caused by LAST are based on the local anesthetic binding site, ion channel inhibition, signaling pathway modulation, or enzymes involved in metabolism.^4–6^ Sodium channel blockade in the CNS and CV system is associated with the main deleterious effects of lidocaine. Blockade of these receptors causes membrane action potential destabilization. CV toxicity occurs because of conduction abnormalities and myocardial dysfunction. CNS toxicity arises due to the modulation of inhibitory pathways in the brain, leading to excitatory clinical features such as sensory or visual changes, muscular activation, and seizure.^4–6^

Lidocaine is 90% metabolized by the cytochrome P450 system via the liver, with CYP1A2 and CYP3A4 specifically having the highest risk of drug interactions. The risk of toxicity is higher when other patient medications are processed the same way and inhibit the enzyme system, slowing down the rate of lidocaine metabolism.^7^ Commonly used substrates that are inhibitors of CYP1A2 and CYP3A4 include antibiotics, antidepressants, antihistamines, and cholesterol-lowering medications.^8^

Additionally, lidocaine metabolism may be affected by other factors, including aging, reduced hepatic blood flow, obesity, and heart failure.^9^ Non-medication inhibitors of CYP1A2 and CYP3A4 include caffeine,^10^ grapefruit juice,^11^ turmeric,^12^ star fruit,^13^ ginkgo biloba,^14^ and cannabidiol.^15^

When metabolism inhibition is not a factor, the lidocaine metabolites monoethylglycinexylidide and glycinexylidide have been shown to exhibit anti-arrhythmic activity and have been associated with some of the adverse effects typically seen with lidocaine therapy.^8^

When used subcutaneously, lidocaine is slowly absorbed into the general circulation as both an unbound component and a component bound to plasma proteins. There is a proven link between lidocaine plasma density and symptoms of toxicity. Unfortunately, we were unable to obtain a lidocaine serum level for this patient. By the time the clinical team had recognized that level measurement may have been helpful, the blood drawn had already been discarded **(Table 1)**.

## Discussion

Though subcutaneous administration of lidocaine can provide excellent pain control, practitioners need to be aware of the risks of CNS toxicity, especially in the context of procedures in vascular areas.

Over the past 30 years, pacemaker placement has become more common as an outpatient procedure with regional anesthesia versus previously utilized inpatient placement with general anesthesia.^3^ Though this is generally recognized as safe and effective, practitioners must be cognizant of the risks of local anesthetics.

Patients are not “one size fits all,” and amounts of anesthetic used must be tailored to their body habitus. The American Society for Regional Anesthesia and Pain Medicine recommends a maximum dose of 5 mg/kg of lidocaine without a vasoconstrictor.^4–6^ In this case study, the patient received approximately four times the maximum dose of subcutaneous lidocaine, and this supratherapeutic dose likely contributed to the development of LAST syndrome. In this case, symptoms resolved within 3–4 h; however, anesthetic toxicity can result in death if not recognized and treated promptly. When a procedure requires more “tries”—in this case, multiple attempts at vascular access due to difficulty with guidewire feeding—the provider must be aware of the total amount of anesthetic used.

Medication review and/or integration of a pharmacist in the treatment team may assist with the identification of LAST. The patient presented was on several medications known to have negative drug–drug interactions with lidocaine. Although these drug interactions are not likely to occur at normal intradermal anesthetic dosages, if additional local anesthetic was required, potential drug interactions should be considered. In our patient, several medications could have played a role in his clinical status. First, bupropion rarely causes seizures.^16,17^ The risk increases when combined with other medications that may lower the seizure threshold, such as systemic exposure to lidocaine.^16,17^ Metoprolol can also increase lidocaine levels due to the potential modulation of lidocaine metabolism from reduced cardiac output and outflow to hepatocytes.^18^ Lastly, divalproex can treat seizures and modulate the seizure threshold. Considering the patient’s past medical history, divalproex was being taken for mood augmentation given his history of anxiety, traumatic brain injury, and migraine headache management.^19^ In addition, both medications may cause methemoglobinemia, a rare condition that leads to oxygen deprivation due to a reduced oxygen-carrying capacity of hemoglobin in the blood.^19^

All things considered, these potential drug interactions likely did not contribute to the patient’s clinical deterioration. The dosages of bupropion and metoprolol mentioned in this case traditionally would not result in supratherapeutic concentrations that lead to the clinical deterioration seen here. We were lacking serum level measurements of divalproex and lidocaine due to a delay in recognizing LAST as the most probable diagnosis in our differential by the treatment team.^16–19^

Health care practitioners must also be prepared to recognize and treat LAST syndrome. As LAST syndrome is rare (recent estimates range from 1–1000 uses of local anesthetic to 1–10,000 uses of local anesthetic), many practitioners have never treated or even witnessed this acute decompensation event.^20^ Further complicating the issue, symptoms of LAST vary greatly and so there are many signs and symptoms that may present as insignificant or mirror other medical events such as a stroke. Finally, while LAST often appears within minutes of administration of local anesthetic, delayed toxicity has also been witnessed. In a paper published by Weinberg et al., in conjunction with the Anesthesia Patient Safety Foundation, the authors recommended that any CNS or CV signs should be viewed as possible symptoms of LAST syndrome until proven otherwise **(Table 2)**.^20^

**Figure 1:**
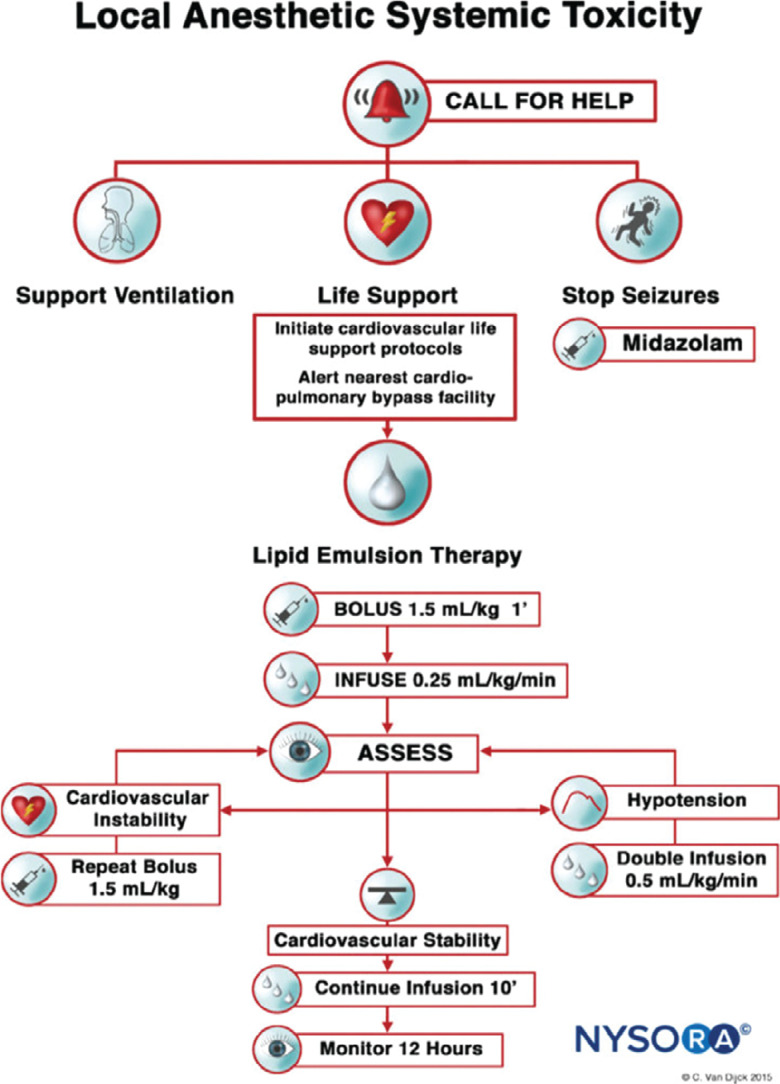
Recommended management of local anesthetic systemic toxicity. Source: NYSORA.COM. Reproduced from NYSORA^22^ with permission.

Therefore, it would benefit health care workers if they receive training in the recognition and management of LAST syndrome, as its treatment is different from traditional cardiac-based advanced cardiac life support. It is prudent that lipid emulsions are easily accessible for the reversal of lidocaine toxicity, and functional intravenous access should be confirmed in each patient receiving local anesthetics.^21^ The checklist from the American Society for Regional Anesthesia and Pain Medicine provides clear guidance when managing LAST **(Figure 1)**.

## Conclusion

This case study explored LAST syndrome in a patient receiving subcutaneous local anesthetic during the placement of a pacemaker. The purpose of this case study was to stimulate discussion around the use of local anesthetics during outpatient cardiac procedures and recognition and treatment of such symptoms.

## Figures and Tables

**Table 1:** Lidocaine Serum Levels and Associated Symptoms

Blood Serum Level	Symptoms
3–6 μg/mL	Metallic taste, circumoral numbness and tingling, light-headedness
5–6 μg/mL	Slurred speech, visual disturbances
8–14 μg/mL	Loss of consciousness, seizures, cardiac arrest

Adapted from Bill et al.^7^.

**Table 2:** Signs and Symptoms of Local Anesthetic Systemic Toxicity

Presenting Symptoms and Signs		
Prodrome	Major CNS	Major CV
Tinnitus Metallic taste Hypertension Tachycardia	Agitation/confusion Obtundation Seizure Coma	Bradycardia/heart block Hypotension Ventricular tachycardia or fibrillation Asystole

*Abbreviations:* CNS, central nervous system; CV, cardiovascular. Reproduced from Weinberg et al.^20^ with permission from the Anesthesia Patient Safety Foundation.
